# Exploration of Joint Optimization and Visualization of Inventory Transportation in Agricultural Logistics Based on Ant Colony Algorithm

**DOI:** 10.1155/2022/2041592

**Published:** 2022-06-15

**Authors:** Bo Dong, Manzhen Duan, Yinfeng Li

**Affiliations:** College of Civil and Architectural Engineering, North China University of Science and Technology, Tangshan, Hebei 063210, China

## Abstract

The problem of joint optimization of inventory and transportation in agricultural logistics and distribution is a typical logistics problem, but agricultural logistics and distribution also have their own characteristics, such as uneven distribution of outlets, complex road conditions, very many outlets, a single order with few goods but high frequency of ordering, centralized distribution, and unified channels. To promote the sustainable development of the economy, it is necessary to save energy and reduce emissions, and eventually enter a new era of “low consumption, low pollution, and low emissions.” Modern logistics vehicle-scheduling process is complex and changeable, and the existing mathematical methods are not perfect in solving this problem, lacking scientific theory as a guide. The joint optimization problem introduces the inventory change factor on the basis of periodic vehicle path optimization and optimizes the inventory decision and path planning in an integrated manner. As a system to support the logistics industry, the visualized logistics information system is capable of video viewing and querying logistics information. In order to reduce gas emissions and save costs, it is necessary to optimize the transportation link, and the focus of optimization is the route optimization of distribution vehicles. Ant colony algorithm (ACA) is an emerging search and optimization technique, which emerged from the research of ACA. In this study, we study the joint optimization and visualization of inventory transportation in agricultural logistics based on ACA. In addition, the experimental results show that the inventory cost/total cost of improved ACA is 0.006 when the unit mileage transportation cost is 10, and the IBM ILOG CPLEX is 0.031, which is reduced by 0.0025, that is to say, in the case of high inventory cost per unit product, the use of improved ACA can lead to a significant reduction in inventory costs. Therefore, it can realize the whole process of control, traceability, and dynamic optimization to ensure the timeliness of emergency finished food security and provide real-time information for decision-making in command as well.

## 1. Introduction

China's agricultural products are not only rich in resources but also rank steadily among the top in the world in terms of production, but the problem of agricultural logistics has been plaguing agricultural players [1]. The so-called logistics refers to the flow process of material materials in physical form from the place of supply to the place of consumption in social reproduction [2]. Through the study and application of optimal scheduling of logistics transport vehicles, it is possible to improve the economic efficiency of logistics and achieve scientific logistics [3]. In the process of logistics distribution, to reduce resource consumption, it is necessary to start from the distribution path, which is the main basis and ultimate goal of sustainable development [4]. Agricultural product logistics and distribution are the end of frustrated agricultural product logistics, connecting agricultural product logistics centers with the final sales outlets such as various farmers' markets and supermarkets [5]. At the same time, with the development of information technology and the changing needs of e-commerce consumers, e-commerce merchants more urgently need third-party logistics to provide personalized and specialized logistics services [6]. Both merchants and consumers want to visualize the entire logistics service process to improve logistics efficiency, reduce logistics costs, and thus, better meet the needs of consumers [7].

The consumption level of the population has gradually increased, the structure of the agricultural industry has gradually made adjustments, and the circulation and production of fresh agricultural products have gradually increased every year [8]. Therefore, more stringent requirements are put forward for the quality and the safety of products [9]. To improve the operational efficiency of the railroad transport industry, reduce costs and enhance service quality, and achieve industrial upgrading, the active development of modern logistics services is the way to go, and the use of computer technology to realize the informationization of logistics is one of the important means [10]. On the one hand, the rapid development of computers promotes the continuous development of optimization methods, on the other hand, it also makes the engineering optimization problems become larger and larger, and the nature of the optimization objective function becomes more and more complex [11]. How to use scientific and effective methods to optimize inventory transportation to improve the economic efficiency of enterprises under the condition of satisfying the diversified needs of customers is an important issue of concern and research focus in the field of logistics today [12].

Chinese logistics enterprises want to grasp the pulse of the future era and become the world market leader, understand the industry competition, win market exclusivity, and seize market opportunities in order to stand firm in this big market economy [13]. In solving multiobjective optimization problems, since the objectives are often in conflict with each other, there is often no constraint that can satisfy all constraints [14]. Instead, there is a set of Pareto optimal solutions [15]. Therefore, the ACA is introduced into this problem to provide technical measures to build an integrated logistics system, establish a modern scheduling and command system, develop an intelligent transportation system, and carry out modern e-commerce with the help of process simulation technology, in view of the comprehensive, complex, and uncertain characteristics of joint optimization and visualization of inventory transportation in agricultural logistics.

The innovative points of this study are as follows:

1. Considering the influence of freshness input on freshness level and quantitative loss, the functional relationship between freshness and quantitative loss is portrayed.
2. Considering the cost of vehicles, such as fixed cost, refrigeration cost, transportation cost, penalty cost, cargo damage cost, and setting the goal of distribution as the total cost minimization, an optimization model is constructed, i.e., the joint optimization model of inventory transportation based on ACA.
3. To conduct a joint optimization study, inventory level and distribution path are affected by market demand, product deterioration rate, and other factors at the same time, and the cost-minimization path can be planned according to the demand node location and distribution volume.

The research framework of this study contains five major parts, which are organized as follows.

The first part of this study introduces the research background and significance, and then introduces the main work of this study. The second part introduces the work related to the joint optimization and visualization of inventory transportation in agricultural logistics, and the development of an e-commerce platform based on ACA for logistics transportation optimization. In the third part, the establishment of ACA-based inventory and transportation optimization model and the establishment of ACA-based inventory and transportation visualization platform are explained, so that the readers of this study can have a more comprehensive understanding of the idea of ACA-based inventory and transportation optimization and visualization in agricultural logistics. The fourth part is the core of the thesis, which describes the application analysis of ACA in the joint optimization and visualization of inventory transportation in agricultural logistics from two aspects, namely, the improvement of ACA analysis and the improvement of the process analysis of ACA for agricultural logistics. The last part of the thesis is the summary of the full work.

## 2. Related Work

### 2.1. Joint Optimization and Visualization of Inventory Transportation in Agricultural Product Logistics

“Logistics” is an emerging science, and the results of this concept are first used in the Second World War in the United States military logistics supply and achieved success; after, the war has attracted the attention of the economic community and developed. Agricultural product logistics usually covers postproduction processing, storage, transportation, and distribution, etc. Through the coordination and cooperation of producers, warehouses, logistics enterprises, retailers, and other subjects, agricultural products enter the field of consumption and finally realize the value added of agricultural products. For the actual situation of logistics application, providing the optimal path information for logistics distribution management platform and distributors is also a necessary means to improve the efficiency of logistics delivery.

Liu realizes the procurement and distribution of customer products through a single vehicle, uses two stacks for the purpose of product transit in the warehouse, and finally solves the vehicle path problem by an improved branch delimitation method [16]. Teschemacher and Reinhart proposed an ant colony system combined with a forbidden search algorithm to solve heterogeneous vehicle path problems with time windows and multiple products, introducing a strategy of two pheromones to accelerate the learning process of ants [17]. Lu et al. performed the solution by a forbidden search algorithm, in which the execution frequencies of insertion and exchange operations are linked to the neighborhood solution set, following the principle of dynamic adjustment [18]. Wang studied the effect of temperature changes in the quality of frozen vegetables, etc., during cold chain logistics distribution, providing a corresponding theoretical basis for reducing the losses in the distribution process of agricultural products [19]. The Xiong design column generation method solves the vehicle distribution problem for larger demand conditions, where the pricing problem is determined by a local search algorithm [20].

The joint inventory-transport optimization problem is an organic combination of inventory decision and path planning, which extends the joint inventory-transport optimization problem that belongs to a spatial decision. By taking inventory management, which is a temporal decision, as a prerequisite for joint inventory-transportation optimization, the joint optimization problem solution is also spatiotemporal in nature. In logistics visualization, storage and warehousing of materials are one of the main activities in logistics visualization. Based on the integration of geographic information and supply and demand information in logistics visualization, a more suitable location for the storage of goods is selected, which saves logistics costs.

### 2.2. Optimization of Logistics Transportation Based on ACA

Logistics transportation is an important part of the logistics system, and the optimal scheduling of logistics transportation vehicles is a key part of logistics system optimization and an indispensable and important part of e-commerce activities. The ACA adopts a positive feedback parallel autocatalytic mechanism, which has the advantages of strong robustness, excellent distributed computing mechanism and is easy to combine with other methods, and has demonstrated its excellent performance and great development potential in many complex combinatorial optimization problems.

Zhou proposed an improved ACA combining a stochastic algorithm and a local search algorithm to solve the distribution vehicle-scheduling problem [21]. Yang et al. proposed the idea of multiple ant colonies to solve the vehicle path problem with time windows, where one ant colony is used to optimize the number of distribution vehicles and another ant colony is used to optimize the total distribution distance [22]. Zhao et al. provided a combined general criterion using heuristics. Since the introduction of heuristics has received better results for the improvement of the performance of global optimization methods, finding better heuristics with more generality is a more researched development direction, especially in metaheuristic algorithms [23]. Liang proposed a saving ACA to solve the vehicle path problem, and the algorithm proposed the probability of attractiveness based on the saving value and then introduced the attractiveness into the state transfer probability [24]. Pei proposed a hypercube framework ant colony optimization algorithm, which restricts the pheromone between 0 and 1 and improves the pheromone update rule [25].

A variety of artificial intelligence algorithms such as evolutionary algorithm, particle swarm optimization, and ant colony algorithm are widely used in machine learning, process control, economic prediction, engineering optimization, and other fields. How to apply these methods to solve complex multiobjective optimization problems with multiple constraints has become a hot topic of research in this field. In the field of customer-centric logistics services, ACA is an indispensable tool by which the provision of integrated and comprehensive logistics management services can be carried out. Using this tool, resource integration can be maximized. Using this means well, it is possible to increase the social and economic benefits well.

## 3. Joint Optimization and Visualization of Inventory Transportation in Agricultural Product Logistics Based on ACA

### 3.1. Establishment of Joint Optimization Model of Inventory Transportation Based on ACA

**Figure 1:**
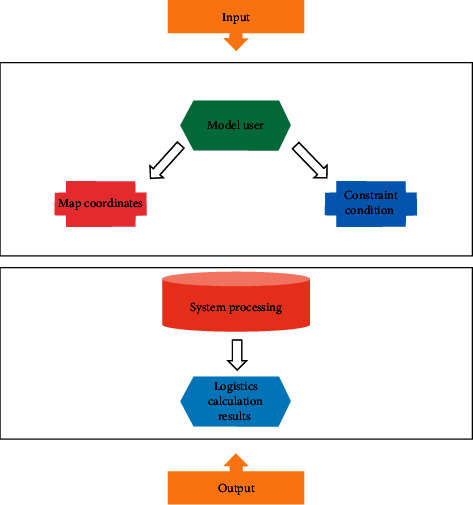
Data flow diagram.

The agricultural logistics system is a continuous and complete chain composed of several “nodes,” including agricultural producers, buyers, distributors, retailers, logistics distribution and storage enterprises, and other logistics entities [26]. The transportation process is carried out between the various nodes and facilitates the downstream flow of agricultural products [27]. After inputting the relevant information, the system is allowed to prioritize the distribution and the results are shown in the data flow diagram in Figure 1.

**Figure 2:**
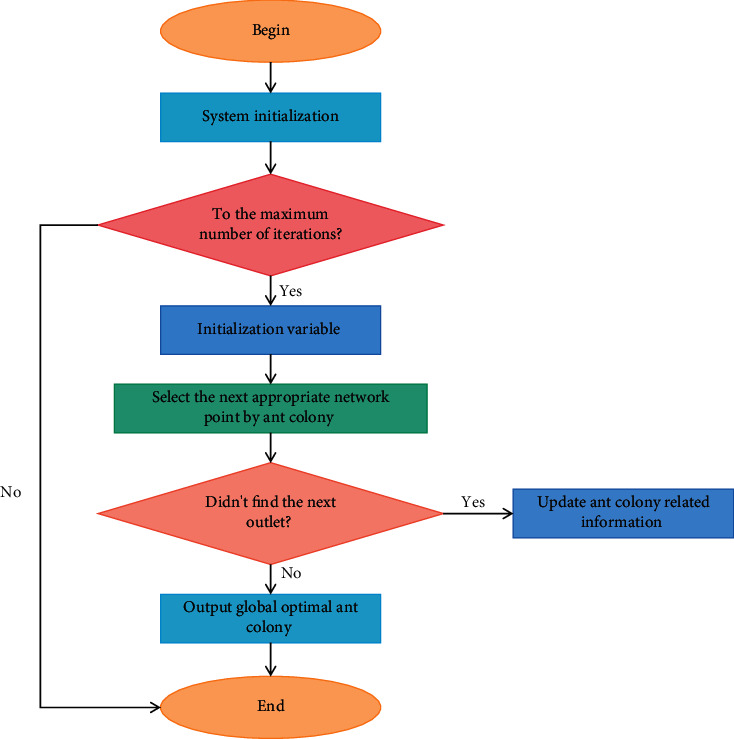
Flowchart of improved ACA.

First, the penalty function is established. The common feature of the penalty cost is that the more the vehicle arrival time deviates from the constraint time window, the higher its penalty cost. Therefore, we assume that the penalty cost linearly increases to simplify the problem. Using Baidu Telematics technology, we call the API interface of the Baidu service to obtain the information on driving route, driving distance and duration between distribution centers and retail merchants, and retail merchants and retail merchants, and write them into the basic information database. The information is initialized (including the number of ants, the amount of information, the maximum number of iterations, etc.), and the initial number of iterations is set to 1. The improved ACA process is illustrated in Figure 2 below.

According to the vehicle loading status, it is divided into the full-load problem and non-full-load problem. Full-load problem means that the freight volume is greater than the capacity of one vehicle, and multiple transport vehicles are required to complete all tasks [28]. Freshness loss and volume loss of fresh agricultural products simultaneously occur, i.e., after a period of time, fresh agricultural products no longer have the initial freshness. For example, water loss from vegetables and fruits, or waste from poor customer-picking behavior can impair product freshness [29]. Therefore, the integration of production, supply, and marketing directly omits the intermediate links and establishes a direct link between producers and retailers by establishing a logistics and warehousing enterprise. This performance evaluation can be used to measure the variation of the distance between adjacent solutions on the resulting Pareto frontier, which is defined as follows:

(1) $$\begin{array}{c} S=\frac{1}{n-1}\sum_{i=1}^{n}{\left(\bar{d}-d_{i}\right)}^{2}. \end{array}$$

Among them,

(2) $$\begin{array}{c} d_{i}=\min_{j}\left(f_{1}{}^{\text{i}}\left(x\right)-f_{1}{}^{\text{j}}\left(x\right)+f_{2}{}^{\text{i}}\left(x\right)-f_{2}{}^{\text{j}}\left(x\right)\right). \end{array}$$

*n*—number of vectors, $\bar{d}$—distance average, and *S*—distance.

Next is the definition of the parameter that each delivery vehicle cannot exceed its rated capacity at one time. In order to improve the utilization of vehicles, the number of vehicles used is minimized through scheduling, while meeting the delivery tasks, and finally minimizing the total cost of vehicle operation. The delivery area is first optimally divided, and then, the delivery routes within the area are optimized. In each step of the path construction, ants follow a probabilistic behavioral selection rule called the random proportion rule to decide which city they will move to next. The probability that an ant located in the current city will choose as the next city is as follows:

(3) $$\begin{array}{c} \left\{\begin{array}{c} {\text{p}}_{ij}^{k}=\frac{\left[\tau_{ij}{\left(t\right)}^{\alpha }\right]{\left[\eta_{ij}\right]}^{\beta }}{\sum_{t\in N_{i}{}^{k}}\left[\tau_{ij}{\left(t\right)}^{\alpha }\right]{\left[\eta_{ij}\right]}^{\beta }},\;j\in N_{i}{}^{k},\\ 0,\;j\notin N_{i}{}^{k}. \end{array}\right. \end{array}$$

*k*—ant, *i*—current city, *j*—next city, and *N*_*i*_^*k*^—feasible neighborhood that can be directly reached.

The next city to be visited is selected based on the state transfer probability and the taboo table changes accordingly [30]. The pheromone that goes to each path changes and an update of the amount of information is required, and if the maximum number of iterations is satisfied, the program is exited, and if not, it returns and continues iterating until the number of iterations is satisfied. The pheromone on all edges is reduced by a fixed size value, and then, each ant adds pheromone to the edge it passes through. This fixed size pheromone is as follows:

(4) $$\begin{array}{c} \tau_{ij}⟵\left(1-\rho \right)\tau_{ij},\;\forall \left(i,j\right)\notin L. \end{array}$$

*ρ*—evaporation rate of pheromone.

According to the transport task that is divided into pure loading problem, pure unloading problem, and loading and unloading mixed problem, the so-called loading and unloading mixed problem is the vehicle in transit during both loading and unloading. On the electronic map, information related to agricultural products (such as retail outlet locations and administrative information) is superimposed. Graphical representation presents a global display effect, convenient for agricultural products business personnel to carry out macroanalysis and decision-making. The volume of cargo transportation tasks is generally larger than one half of the vehicle capacity, or the collection and delivery points of each task are relatively scattered. At this time, the goods are difficult to mix, and the vehicle completes a task and then empties to the next task collection point to complete the next task. After completing the pheromone evaporation step, all ants release pheromones on the side through which it passes the following:

(5) $$\begin{array}{c} \tau_{ij}⟵\tau_{ij}+\sum_{k=1}^{m}\nabla \tau_{ij}{}^{k},\;\forall \left(i,j\right)\notin L. \end{array}$$

∇*τ*_*ij*_^*k*^—the amount of pheromone released by the *k* first ant to its passing edge.

Finally, since the operation of each logistics truck is independent, in order to ensure the “first-come-first-served” scheduling rule of the service, a dynamic time series needs to be established to represent the time series of the boxes available at each depot. When the delivery starts, the Android vehicle terminal calls the remote service interface to read the delivery task information of the associated delivery vehicle, such as delivery serial number, merchant order number, merchant address information, and the total number of goods. In the process of making the delivery, we also have to consider the sorting system in distributing varieties and the balance of the transport vehicle operation. Therefore, each freight task has its own collection point and delivery point, and the vehicle departs from the yard, goes to the collection point of a certain task, loads the goods after transporting to its delivery point to unload that is loading and unloading mixed, and returns to the yard after completing all tasks.

### 3.2. Establishment of Visualization Platform of Inventory Transportation Based on ACA

**Figure 3:**
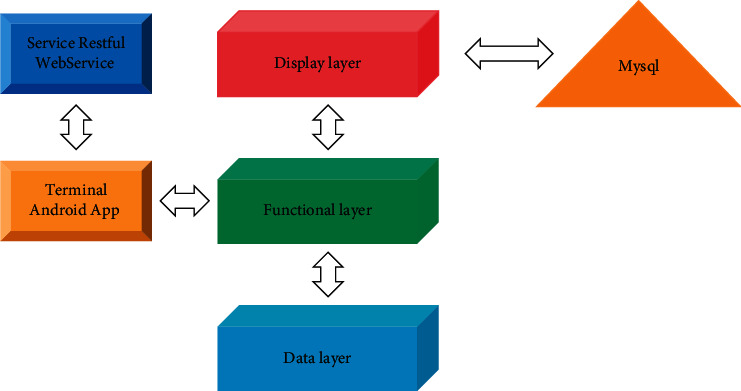
Multilayer architecture technology of the system.

The main components of the visualized finished grain logistics information system include vehicle terminals, communication, and video monitoring center. In transit visualization is mainly to be able to monitor the vehicle's operation status and operation route in the control center. Open-source framework technology is used. The main framework technologies include the following: JSF2, Spring3.0, and JPA. The platform is designed with a three-tier architecture, as shown in Figure 3 below.

First is the display layer, which is used to visualize the data information of the finished grain logistics information system, and clearly shows the business process between the event occurrence and the event completion. We visualize the relationship between data, and the main application technology is the front-end display technology, such as JSP, HTML, and Flash technology. The visualization functions in this project include real-time visualization and monitoring of logistics in holding warehouses, real-time visualization and monitoring of remote logistics, management of refrigerated vehicles, and real-time monitoring of refrigerated vehicles. The distance between the collection point and the distribution center cannot be a straight-line distance (Euclidean distance), according to its actual road conditions is an available formula to calculate the distance between these two points, and the vast majority of roads will be in this mode as follows:

(6) $$\begin{array}{c} d_{i}=\left|x_{i}-x\right|+\left|y_{i}-y\right|. \end{array}$$

Transportation lines and warehouses constitute a logistics network in the logistics system, and warehouses are the nodes of the network, which need to determine the routes according to the nodes and then form the transportation and distribution network. Using a continuous ACA, it first performs a global search of the solution space using a genetic algorithm. Then, ACA is used to locally optimize the results obtained by taking the farthest demand point from the depot as the seed point of the route. The next insertion point is the one with the smallest insertion value according to the nearest-neighbor insertion method. Finally, the generalized savings formula is used to determine the insertion position with the largest savings value, and the selection and insertion steps are repeated. When the path can no longer be expanded, another route is created. The algorithm generally uses the error sum-of-squares criterion function as the clustering criterion function, and the error sum-of-squares criterion function is defined as follows:

(7) $$\begin{array}{c} J_{c}=\sum_{i=1}^{k}\sum_{p\in C_{i}}{\left\|p-M_{i}\right\|}^{2}. \end{array}$$

*p*—sample point and *M*_*i*_—average value of data.

Next is the functional layer: it is mainly the 6 functional modules of the system, including business functions and system setting functions, business functions constitute the thematic framework of the system, and auxiliary functions are used for system setting, etc., which cooperate to complete the realization of the main functions. To realize the visualization function of the system, it is necessary to equip video collection equipment and sensor nodes in key links to collect information in real time. The sparsity of the feasible region is usually measured by the ratio of the feasible region to the whole search space. At the same time, the intensity of the variation of the constraint on the boundary of the feasible region is also greatly related to the determination of the penalty term. In order to meet the supply and demand transportation and distribution needs and ensure economic efficiency, it is necessary to set up a reasonable number of warehouses or other facilities in the identified area, and determine the size and location of each facility and the logistics connections existing between the facilities, etc. The facility location model can better solve these problems. Based on such adjustment rules, the advantage of the local optimal path is appropriately weakened, and the algorithm converges to the local optimal solution by effectively avoiding the information on the local optimal path that is far too large. The space composed of all points on the decision space by the mapping of the objective function is called the objective function space. Its feasible domain is described by the following equation:

(8) $$\begin{array}{c} D_{f}=\left\{F\in {\left[f_{1}\left(x\right),f_{2}\left(x\right),\ldots ,f_{q}\left(x\right)\right]}^{T},\;X\in D_{x}\right\}. \end{array}$$

*n*—*n* dimension decision variable, *x*—decision vector, and *F*(*x*)—target vector.

The decision space refers to the space composed of all the parameters of the objective function that meet the constraint conditions as follows:

(9) $$\begin{array}{c} D_{x}=\left\{\text{X}\in {\text{R}}^{n}|g_{u}\left(x\right)\geq 0,h_{v}\left(x\right)=0\right\}. \end{array}$$

Finally, there is the data layer; the system database table is constructed, including the spatial database and the attribute database; and the data can be added, deleted, changed, and checked. After the data are stored, the picture can be investigated by inquiry, and the monitor can also monitor the status of frozen food at that time. A logistics company has to set up *D* logistics transit points, and it is necessary to consider that these transit points can cover a certain area, and the number of customers at each transit point should be approximately the same level. Using a uniform constant as a posteriori function, this cannot achieve the purpose of distinguishing the superiority from the inferiority. In this study, the dominance factor of CLS will be considered in the heuristic function, and the heuristic information is calculated as follows:

(10) $$\begin{array}{c} \eta_{\left(j,b\right)}=\frac{1+{\text{CLS}}_{jb}.D}{N}. \end{array}$$

*N*—the number of ALS.

At the beginning of the iteration, the pheromone concentration on all paths is set to the upper pheromone limit, which helps to increase the search ability of the algorithm at the beginning and avoid the phenomenon that the initial convergence ability of the algorithm is constrained by the small initial pheromone and the large influence of the posterior factor. The topological nature of the surface corresponding to the objective function, such as under the same constraints, linear or convex function, is easier to solve than the irregular function. The logistics nodes such as warehouse, vehicle dispatching, and demand destination need to be marked on the electronic map with specific geographical locations. The emergency monitoring also needs to obtain various data collected from the vehicle terminals from the system database, including distance, road level, road condition video, and other vehicles and road conditions.

## 4. Application Analysis of ACA in Joint Optimization and Visualization of Inventory and Transportation in Agricultural Product Logistics

### 4.1. Improved ACA Analysis

The ACA can be used to solve TDP and VRP problems and many other problems, and is very scalable.

**Figure 4:**
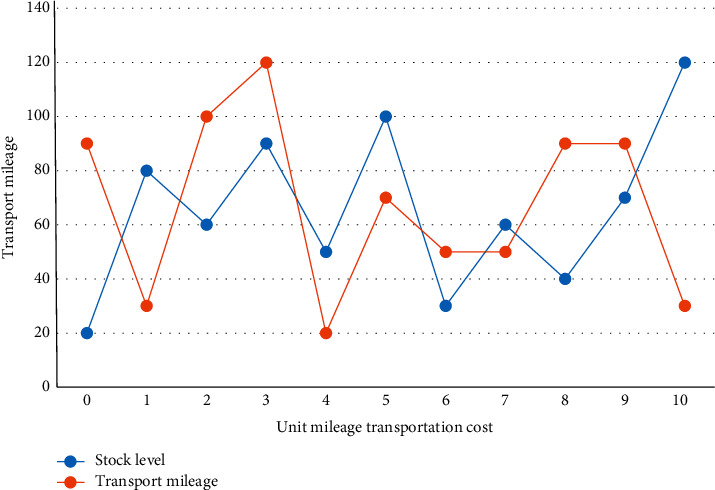
Relationship between inventory level and transportation mileage.

First, we read the set of orders to be delivered on a certain day for agricultural products and obtain the basic information of the associated agricultural products retail outlets, such as number, latitude, and longitude. The retail outlets of agricultural products are equivalent to cities in ACA. From the perspective of logistics cost, we analyze how the research algorithm weighs the relationship between transportation volume and inventory volume, and give the check-and-balance relationship curve between inventory level and transportation mileage, as shown in Figure 4.

The problem model mainly involves a procurement network consisting of several farmers' cooperatives and a super logistics center to fully reduce inventory and transportation costs under the premise of meeting the procurement needs of the logistics center. Due to the asymmetry of market information and the special characteristics of fresh produce, in order to reduce the transaction risk, it is necessary to carefully find cooperative enterprises and supervise the completion of the performance, and if there are too many upstream and downstream partners or the updated information is not timely, it is necessary to pay higher transaction costs. To further test the performance of the algorithm, the study cites the sets of S12T4, S20T21, and S50T21 arithmetic cases and compares the results of the algorithm in this chapter with those of the hybrid genetic algorithm, and the computational results are shown in Table 1.

**Figure 5:**
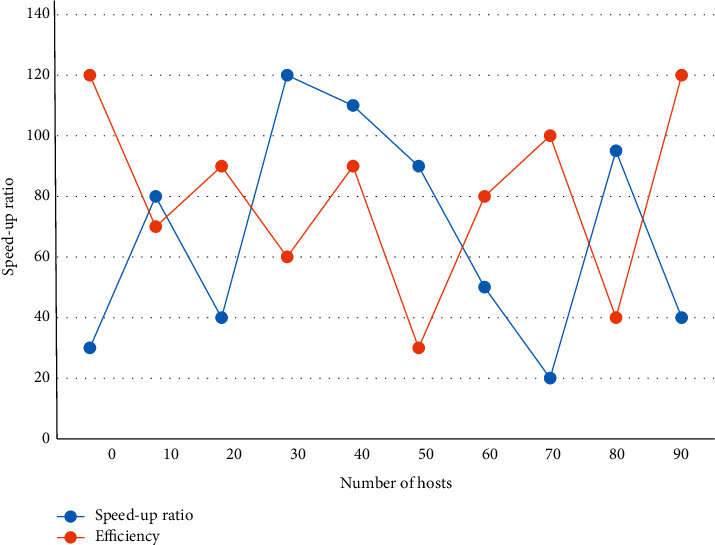
Trend diagram of acceleration ratio of parallel ACA experiment.

It should be emphasized that the main purpose of introducing artificial ants is to allow these ants to find some better routes between the corresponding the two nodes, and by better routes, we mean those routes that choose a node that is closest and takes the shortest time. Replacing a vehicle with an artificial ant to serve a customer point returns to the distribution center when the next customer point to be served would cause the total amount of the shipment to exceed the car's capacity or cause the distance to exceed the maximum distance traveled at one time. Indicating that this vehicle completes this transport, the vehicle then departs to serve the remaining customers until all customer points have been served at one time. The acceleration ratio trend graph for the parallel ACA experiment is shown in Figure 5.

**Figure 6:**
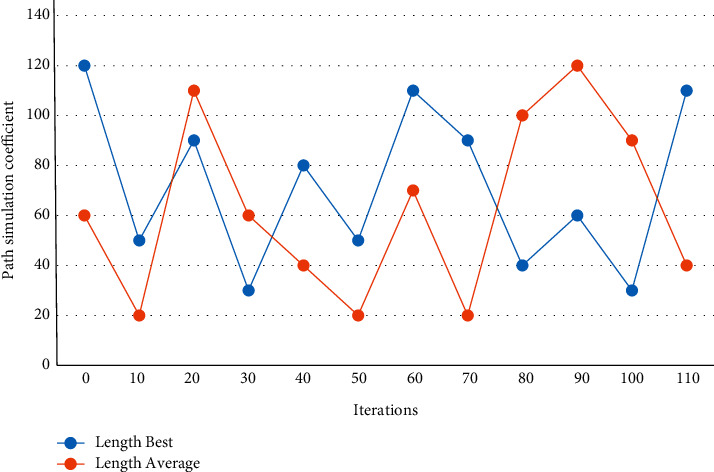
Adaptive ACA path simulation diagram.

Second, the global optimal ratio of vehicle loading to vehicle capacity, the global optimal ratio of individual vehicle loading to vehicle capacity, and the default individual vehicle loading to vehicle capacity are set. Unlike the simple path-planning problem, the model takes into account conditions such as product demand and inventory of each product in the logistics center, in addition to the transportation distance. The length of the path determines the probability size of the ants in choosing the next node and the concentration of pheromones left between two nodes. In order to keep a balance between “exploration” and “utilization” and to avoid stagnation while the algorithm has a strong search capability, the adaptive ACA uses adaptive pseudorandom rate selection rules. Due to a large amount of cargo for each task, each vehicle can only go to one task, in which case, the vehicle returns to the yard after loading or unloading directly from the yard to the task point. Let the pheromone parameter = 1, heuristic factor = 5, and information evaporation factor = 3, the adaptive ACA path simulation diagram is obtained, as shown in Figure 6.

Finally, the suitable outlet is one of the next outlets set reachable by the ant's current outlet, and the cargo volume of this outlet is less than the remaining load volume of the ant. To increase the vehicle full-load rate, the logistics center purchases products in each procurement period by LTL transport, i.e., a single vehicle visits each cooperative in sequence and without considering the maximum load of the transport vehicle. However, due to the excessive growth of pheromones on some edges, this strategy may lead to a stagnation phenomenon where all ants search for the same path, and although the paths on which these edges are located may be better, they are often not the optimal ones. Therefore, penalties are considered for deviations from customer requirements, and customer losses due to deliveries outside the service time window are included in the penalty cost. The volume of freight task is small compared with the vehicle capacity, which generally means less than one-half of the vehicle capacity, and some tasks have concentrated collection and delivery points. We consider that one vehicle needs to load and unload goods at multiple assembly points. In order to achieve the above purpose, the goods can be mixed and loaded first, and then unloaded at several corresponding delivery points.

### 4.2. Process Analysis of Improving ACA in Agricultural Product Logistics

In the discrete space optimization problem, the travel of ants is achieved by jumping on the set of points in the discrete solution space, i.e., moving directly from one node to another node in the solution space. The distribution customers are mostly retailers, which belong to the end of the supply chain and are responsible for the sales of agricultural products. In the case of multiple yards, the transportation vehicles depart from multiple yards to perform tasks at multiple task points, which is the general transportation problem in operations research. Therefore, the improved ACA defines an activity range for the ants, which can only be within the radius of the ants, in order to increase the search range of the algorithm; as the number of iterations increases, the range of random perturbations can be linearly reduced to enhance the local convergence ability of the algorithm.

**Figure 7:**
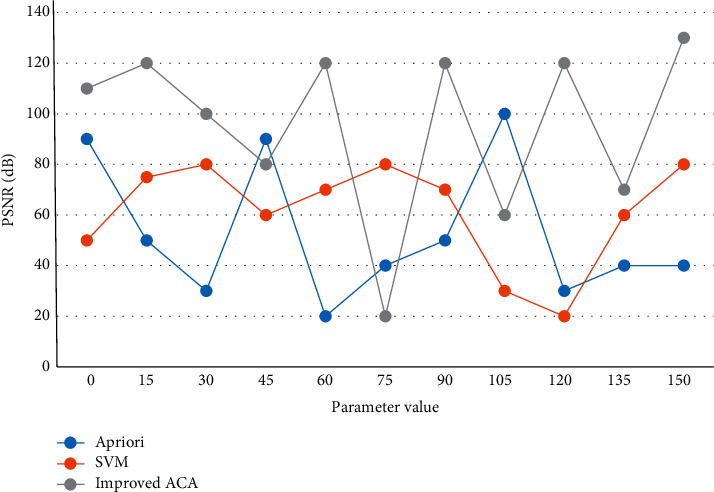
Simulation comparison of three different algorithms.

First, it is necessary to check whether there are goods placed on top of the exit tables of the 10 lanes and also to check the backlog signs of the transports to see whether the transports are in a waiting state. Similar to real ants that leave pheromones on the road, these electronic ants convert the information they collect, i.e., the speed and congestion of the route into electronic codes that are left on the road they pass. The existing pheromones on the road start to “evaporate,” i.e., decrease, in proportion to a fixed parameter: each ant then adds new pheromones to the road it passes. The constraints of the problem are expressed in the form of states, and special operators are designed so that the solutions represented by the states remain viable during the search process. Since the update of the pheromone directly affects the selection probability, if the update of the pheromone is made more diverse, the selection of paths will be diversified. Therefore, the simulation results of the apriori algorithm, SVM algorithm, and improved ACA are compared, as shown in Figure 7 below.

Next, the information obtained in the first step is stored inside the *D*_*s*_ sequence. Based on the data inside the *T*_*S*_ sequence and the cargo priority *Y*_*s*_ sequence, the cargo priority size of these 10 lanes is found. For the dense distribution near the optimal point, using the local strategy can search only the subregion where the optimal point is located, which will be more effective and fast to search for the new minimum value. For the yard, logistics information technology allows freight forwarders to quickly and comprehensively grasp the nature, quantity, and flow of LTL cargo, so as to develop a reasonable loading and unloading operation plan, to better implement the principle of “sitting over the main, landing as a supplement.” At the same time, as the key facilities distribution information in the visualized grain logistics information system, such as finished grain storage warehouses, vehicle dispatching centers and finished grain demand destinations are indispensable databases for spatial query and route planning analysis, and are also the basic attributes of the finished grain logistics information system. Therefore, the improved ant algorithm applied to the path scheduling of conveying trolleys can find out the shortest path, and after the shortest path encounters the phenomenon of blockage, it can promptly discover and bypass this blockage break to find another suboptimal route. To further illustrate the effect of considering inventory on path planning in a finite cycle, let the transportation cost per unit mile be 10, the algorithm is optimized by IBM ILOG CPLEX and improved ACA, respectively, and the results are shown in Table 2.

The inventory cost/total cost of the improved ACA is 0.006 for a unit mile transportation cost of 10, compared with 0.031 for IBM ILOG CPLEX, a reduction of 0.0025, i.e., it shows that the inventory cost can be significantly reduced by using the improved ACA in the case of high inventory cost per unit of product.

**Figure 8:**
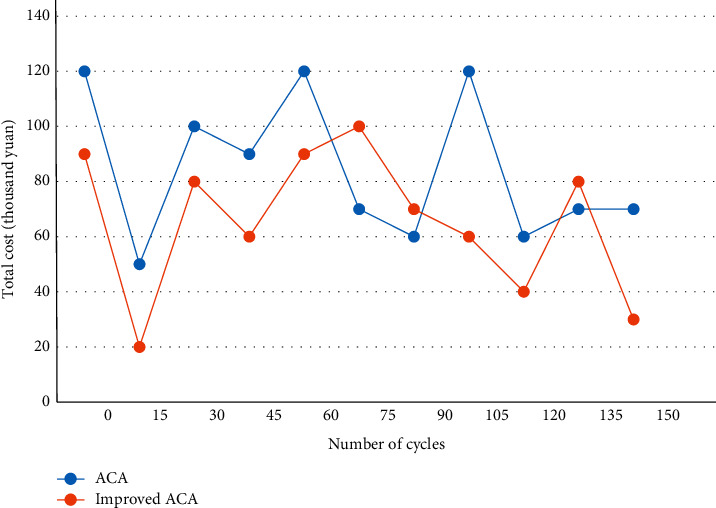
Comparison chart of total cost convergence.

The problem is solved with the basic ACA and the improved ACA, respectively, and run in the MATLAB environment to obtain a comparison graph of the total cost convergence of agricultural logistics distribution, as shown in Figure 8.

Finally, the two lanes with the highest priority are obtained, and they are prioritized for transport pickup. We make the network signal propagate along the line with the highest degree of electronic pheromone reinforcement. By using a positive feedback mechanism to enhance the attractiveness of paths with ant paths to ants in subsequent iterations, the elite ant system additionally enhances the strength of information on contemporary optimal paths. However, because of this, the algorithm is prone to stagnation after a certain number of iterations and does not continue to explore other new solutions. Therefore, the ants deliver to the customer point from the yard and then return to the yard to form a delivery route after the delivery is completed, and all the delivery routes are added together to form a complete delivery route. The constraints are not considered in the coding process, and the solution abandonment is decided by checking the feasibility of the solution in the search process.

## 5. Conclusions

The development of modern logistics technology and the widespread use of the automated three-dimensional warehouse, resulting in an automated three-dimensional warehouse in the joint optimization of inventory transportation and scheduling problems, have become the bottleneck in modern logistics technology that has yet to be solved. The modern economy is a more important part of modern logistics, the development of modern logistics to improve the country's economy, optimize the system configuration, save time costs, etc. is of great significance. In addition, the traditional information system of finished grain cannot meet the needs of users, and the visualization of the information system is getting more and more attention. The ACA can complete the construction of a global solution by local solution with the help of positive and negative feedback function; in addition, it can prevent the algorithm from entering the local optimal mode with the help of negative feedback function. Therefore, in this study, based on the analysis of the current development of China's agricultural logistics system, the research focuses on the inventory and transportation aspects to improve the comprehensive decision-making level, and proposes the joint optimization of inventory and transportation with the “efficiency backward” relationship. This study introduces ACA into logistics vehicle scheduling and explores the joint optimization of inventory and transportation of transportation vehicles by using ACA to pursue the optimal solution with the lowest total cost and the greatest total benefit of the transportation system. Therefore, the study of joint optimization and visualization of inventory transportation in agricultural logistics based on ACA takes into account the constraints closer to the reality and has the advantages of simplicity, intuition, easy-to-understand, and easy-to-design algorithms to solve and strengthen expandability.

## Figures and Tables

**Table 1:** Comparison of algorithm results.

Example	S12T4	S20T21	S50T21
Algorithms in this chapter	19.172	27.172	32.241
Hybrid genetic algorithm	15.263	19.761	22.654

**Table 2:** Optimization results of one-to-many distribution model.

	IBM ILOG CPLEX	Improved ACA
*H*	6	10
Optimal solution	1425	3461
Inventory costs/total cost	271/8815	52/8900

## Data Availability

The dataset can be accessed upon request.
